# Psychological Distress Among Infertility Patients: A Network Analysis

**DOI:** 10.3389/fpsyg.2022.906226

**Published:** 2022-06-28

**Authors:** Danfeng Cao, Caifeng Bai, Guoxiang Zhang

**Affiliations:** ^1^Department of Obstetrics, The First Affiliated Hospital of Shandong First Medical University and Shandong Provincial Qianfoshan Hospital, Jinan, China; ^2^School of Nursing, Ningxia Medical University, Yinchuan, China

**Keywords:** psychological distress, infertility, network analysis, depression, anxiety

## Abstract

**Background:**

Psychological distress is common among infertility patients. Total scale scores are often used to represent the severity of anxiety, depression, or stress, which ignores important differences between specific symptoms, and relationships between symptoms. This study aimed to identify patterns of psychological distress experienced by infertility patients and to identify the most central symptoms of anxiety, depression, and stress.

**Method:**

From June to September 2016, 740 infertility patients were included in this cross-sectional study. Infertility patients were asked to complete the Generalized Anxiety Disorder-7, Patients Health Questionnaire-9 (PHQ-9), and Fertility Problem Inventory. Network analysis was used to examine the patterns of psychological distress in infertility patients and to test the most central symptoms of anxiety, depression, and stress.

**Results:**

Restlessness was the most central symptom in infertility patients. “Feelings of guilt” had the highest strength among PHQ-9 symptoms. “Relationship concern stress” and “sexual concern stress” had the strongest connections in the network. Stability estimation indicated that the order of node strength centrality was more stable than the order of closeness and betweenness (the CS-coefficients were 0.75, 0.13, and 0.67, respectively). In addition, network structure and global strength were invariant across gender.

**Limitations:**

The cross-sectional design did not permit identification of causal relationships. Patients in this study were recruited from one reproductive hospital; especially, most patients had low socioeconomic status, which limits generalizability of the findings.

**Conclusion:**

This study reinforces the need to better understand the underlying causes of psychological distress in infertile patients. A more detailed investigation of the relationship between these symptoms could provide information for psychosocial interventions aimed beyond “alleviating psychological distress.” We should consider the individual psychological symptom pattern and its potential causes in infertility patients instead of assuming a consistent psychological distress structure.

## Introduction

Infertility is recognized as a global public health problem by the World Health Organization (Macaluso et al., 2010). Global estimates suggest that one in six couples has been affected by infertility at least once in their lives. In China, the prevalence of infertility was as high as 25.0% (Zhou et al., 2018). The experience of infertility and involuntary childlessness is devastating for infertile individuals because of infertility itself, intrusive medical procedures, high costs, and unpredictable outcomes (Verhaak et al., 2007; Bai et al., 2019a). Anxiety, depression, and stress are the most common forms of psychological distress among infertile patients (Volgsten et al., 2010; Omani-Samani et al., 2018; Maroufizadeh et al., 2019), which are associated with poor marital quality and quality of life, lower compliance with fertility treatments, and adverse IVF outcome (Wang et al., 2007; Crawford et al., 2017; Haimovici et al., 2018). Thus, psychological distress among infertility patients warrants considerable attention.

The most common theories used in studying these kinds of distress and their co-occurring symptoms are the common cause theory and latent variable perspective. In the common cause theory, symptoms co-occur because of an underlying common cause (Beard et al., 2016). For example, in depression among infertility patients, it is assumed that symptoms such as sleep difficulty, sad mood, and fatigue are caused by depression, just as pneumonia causes fever and dry cough. Likewise, in the latent variable perspective, it is assumed that symptoms, such as sleep difficulties, sad mood, and fatigue, are caused by an underlying latent variable that represents depression, while symptoms, such as nervousness, worry, and fear, are caused by an underlying latent variable that represents anxiety. Thus, the total scale scores have often been used to represent psychological distress severity, assuming that symptoms are interchangeable indicators of the same underlying conditions, and therefore can be summed to create a total score (Fried and Nesse, 2015; Beard et al., 2016). However, the common cause theory may ignore important differences between specific symptoms, and relationships between symptoms (Beard et al., 2016). Psychological symptoms are often not the result of common causes, but cause each other (Cramer et al., 2010).

Network analysis is a novel way to describe symptom–symptom connections. In network analysis, symptoms are part of a dynamic symptoms network that produce, sustain, and underlie mental disorders (Levinson et al., 2017). For example, in depression among infertility patients, sleep difficulties might be highly related to fatigue, not because they come from the same latent variable that represents “depression,” but because poor sleep directly influences fatigue. Network theory has not yet been applied to psychological distress among infertility patients. The application of network theory may clarify some crucial issues of psychological distress in infertility patients. First, network analysis could describe and quantify the symptoms that are most central in infertility-related psychopathology. The core symptoms are highly central and might play an important role in the network. Thus, interventions should be targeted to these core symptoms (Borsboom, 2017; Levinson et al., 2017). Second, network analysis allows researchers to test how such symptoms influence one another. Interventions aimed at reducing specific core symptoms (symptoms associated with most other symptoms) should also theoretically also reduce related symptoms (Levinson et al., 2017). Third, addressing core co-occurrence symptoms may disrupt or weaken the circulation or connections between multiple psychopathological symptoms, such as depression and anxiety (Borsboom, 2017; Levinson et al., 2017). Identification of these networks may eventually generate more fine-grained interventions that target the core symptoms of the network.

In summary, this study is the first to apply network theory to psychological distress including anxiety, depression, and stress among infertility patients. Thus, this study aimed to examine the patterns of psychological distress in infertility patients, and to test the most central symptoms of anxiety, depression, and stress.

## Materials and Methods

### Participants

From June to September 2016, a sample of 740 infertility was examined in a reproductive medicine center of a hospital in Ningxia Province, China. The following inclusion criteria were used: (a) not been pregnant for at least 12 months prior to study participation and (b) at least junior high school. The following exclusion criteria were applied: (a) current mental disorders or organic-brain disorders such as dementia or delirium and (b) couples undergoing cycles with gamete donation. Patients in this study filled the questionnaires guided by the researchers. Ethical approval was obtained from the ethical review board of the Ningxia Medical University.

### Measures

#### Anxiety

Anxiety was assessed by the Generalized Anxiety Disorder-7 (GAD-7; Spitzer et al., 2006) questionnaire, which a 7-item self-report scale, also based on DSM-5 criteria. The item scores range from 0 to 3. The higher item score represents more anxiety symptom. GAD-7 consists of seven items: (a) nervous or anxious; (b) uncontrollable worry; (c) worrying too much; (d) trouble relaxing; (e) restlessness; (f) irritable; and (g) afraid something awful might happen. The validity of the Chinese version of GAD-7 has been demonstrated in China (Gong et al., 2021). The Cronbach’s *α* of the GAD-7 in this study was 0.909.

#### Depression

Depression was measured by the Patients Health Questionnaire-9 (PHQ-9; Kroenke et al., 2001). Each item is assessed on a four-point Likert scale ranging from 0 to 3. The higher item score represents more depression symptom. The PHQ-9 consists of nine items: (a) anhedonia; (b) depressed or sad mood; (c) sleep difficulties; (d) fatigue; (e) appetite disturbances; (f) feelings of guilt; (g) concentration difficulties; (h) psychomotor agitation or retardation; and (i) thoughts of death. The validity of the Chinese version of PHQ-9 has been demonstrated in China (Wang et al., 2014). The Cronbach’s *α* of the PHQ-9 in this study was 0.850.

#### Fertility-Related Stress

Fertility-related stress was assessed by the Fertility Problem Inventory (FPI; Newton et al., 1999). The FPI is a self-rating scale consisting of 46 items that form five subscales: social concern, sexual concern, relationship concern, need for parenthood, and rejection of childless lifestyle. The participants respond on a 6-point Likert scale ranging from 1 to 6. The higher item score represents more fertility-related stress. The validity of the Chinese version of FPI has been demonstrated in China (Peng et al., 2011). The Cronbach’s *α* of the five facets of the Chinese version were 0.76, 0.75, 0.73, 0.77, and 0.82, respectively.

### Statistical Analysis

#### Network Estimation

We used the R package qgraph to estimate the network through a graphical gaussian model (Lauritzen, 1996). In network models, individual symptoms are represented as “nodes,” and “edges” between nodes can be understood as partial correlations, which were considered as the associations between two nodes when controlling for all other nodes in the network. Notably, for PHQ-9 and GAD-7, every item represents one symptom, thus the use of total scores ignores important differences between specific symptoms, and relationships between symptoms. For infertility-related stress, not every item represents one aspect of stress, but each dimension represents one aspect of stress. Thus, for infertility-related stress, the use of dimension scores is a more reasonable approach. Thus, seven symptoms of depression, seven symptoms of anxiety, and five dimensions of infertility-related stress were included in the network analysis. We conducted the least absolute shrinkage and selection operator (LASSO) method to control for spurious associations that might be caused by sampling error (Friedman et al., 2008; Epskamp et al., 2018). LASSO is an L1-regularization technique that accurately reduces small correlations to zero by assigning penalties to limit the number of false (false positive) edges, resulting in a more interpretable and sparser network (Friedman et al., 2008; Epskamp et al., 2018).

#### Centrality Estimation

We calculated the following indexes of node centrality to assess the importance of nodes (Levinson et al., 2017): *node strength*, *betweenness*, and *closeness*. *Node strength* refers to the sum of the direct connections of a node. *Betweenness* measures the number of times in which a node lies on the shortest path between two other nodes. *Closeness* is the inverse of the sum of the shortest paths to all other nodes, measuring how fast a node can be reached from the other nodes. High values indicate a high level of centrality.

#### Accuracy and Stability Estimation

We estimated network accuracy and stability using the R-package bootnet (Epskamp et al., 2018). First, we bootstrapped the 95% CI around each edge in the network to estimate the accuracy of the edges. Larger edge weight CIs indicate lower accuracy. The R-package bootnet also allows one to test whether certain edges are stronger than others. Only the edges that prove to be significantly stronger than most other edges can be interpreted as such. Then, we evaluated the stability of centrality using a bootstrap person-dropping procedure that provides a central stability coefficient (CS-coefficient). It is recommended that the CS-coefficient should be at least 0.25, and preferably above 0.50 (Epskamp et al., 2018).

#### Sensitivity Analysis

Lastly, we compared the difference in network structures between infertile women and infertile men using the Network Comparison Test. The significance of group differences in the following parameters can be tested by reference distribution (Van Borkulo et al., 2017, manuscript submitted): (1) invariant network structure, which concerns the structure of the network as a whole, (2) invariant edge strength, that is, the difference in strength of a specific edge of interest, and (3) invariant global strength, which is the sum of all node strengths.

## Results

### Sample Characteristics

Among 741 patients, 51.4% (*N* = 380) were women and 48.6% (*N* = 360) were men. Most participants were Han Nationality (76.8%), had a junior high or high school education (73.0%), had a monthly family income of less than 3,000 Chinese yuan (72.4%). The mean duration of infertility was 3.03 years (SD = 2.66). The mean number of treatments was 3.92 (SD = 1.55). Other sample characteristics are available in Bai et al. (2019b).

### Network Structure

**Figure 1:**
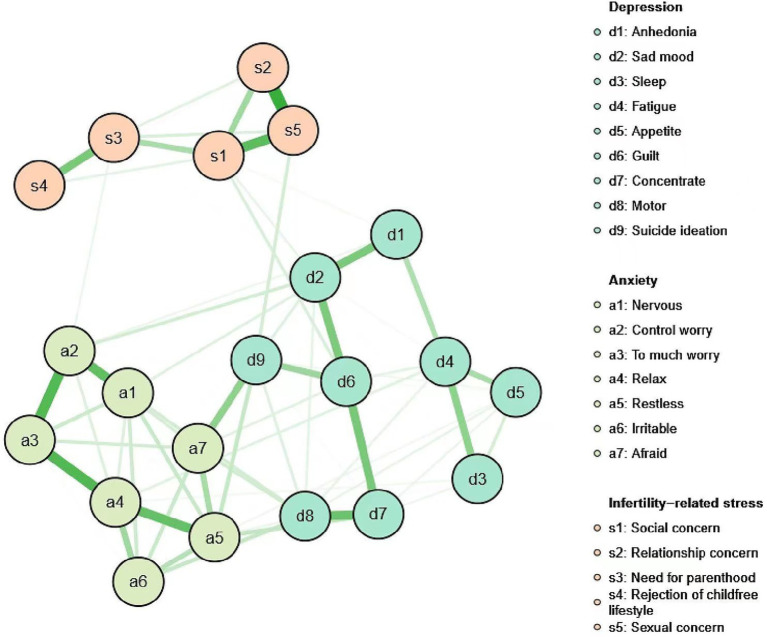
Network containing the nine PHQ-9 depression symptoms, seven GAD-7 anxiety symptoms, and five FPI infertility-related stress symptoms. PHQ-9, Patient Health Questionnaire-9; GAD-7; Generalized Anxiety Disorder-7; and FPI, Fertility Problem Inventory.

**Figure 2:**
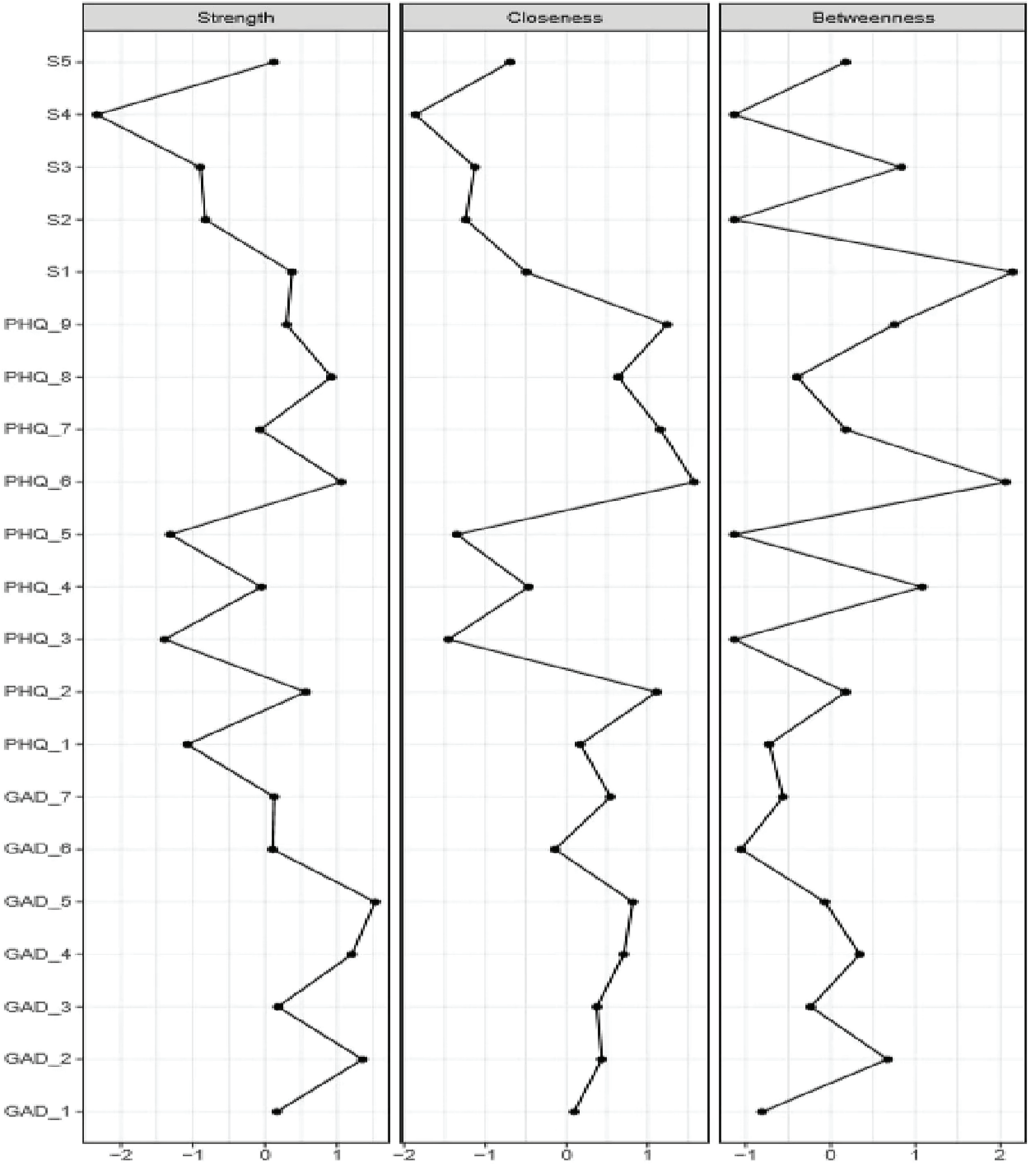
Betweenness, closeness, and node strength centrality estimates for nine PHQ-9 depression symptoms (PHQ_1–PHQ_9), seven GAD-7anxiety symptoms (GAD _1–GAD_7), and five FPI infertility related stress symptoms (s 1–s5). PHQ-9, Patient Health Questionnaire-9; GAD-7; Generalized Anxiety Disorder-7; FPI, Fertility Problem Inventory; sl, Social concern; s2, Relationship concern; s3, Need for parenthood; s4, Rejection of childless lifestyle; and s5, Sexual concern.

Symptoms, sample means, and standard deviations are shown in Table 1. The network is depicted in Figure 1; the Centrality indices are shown in Figure 2. About 48.1% of all network edges were set to zero. Overall, symptoms were positively connected within the network. Significance tests of edge weight differences (see Supplementary Figure 1) showed that the strongest edges in the network were between items from the same scale. The top edge was between the infertility “relationship concern stress” and “sexual concern stress,” which was significantly different from nearly all the other edges in the network. In addition, the GAD-7 items “Worry too much (GAD_3)” and “Trouble relaxing (GAD_4),” “Uncontrollable worry (GAD_2)” and “Worry too much (GAD_3),” “Nervous or Anxious (GAD_1)” and “Uncontrollable worry (GAD-2),” “Trouble relaxing (GAD_4)” and “Restlessness (GAD_5),”the FPI items “Social concern” and “sexual stress,” “Need for parenthood” and “Rejection of childfree lifestyle,” and the PHQ items “Concentration difficulties (PHQ-7)” and “Psychomotor agitation/retardation (PHQ_8),” “Feelings of guilt (PHQ_6)” and “Concentration difficulties (PHQ_7),”“Anhedonia (PHQ_1)” and “Depressed or Sad mood (PHQ_2),” “Sleep difficulties (PHQ_3)” and “Fatigue (PHQ_4)” were connected and significantly different from about two-thirds of the other edges. Additionally, the PHQ item “Suicide ideation (PHQ_9)” and the GAD item “Afraid (GAD_7),” the PHQ item “Feelings of guilt (PHQ_6)” and “Thoughts of death (PHQ_9),” “Sexual concern stress” and “Relationship concern” were connected and significantly different from half of the other edges.

**Table 1:** Symptoms, sample means, standard deviations, and distribution.

Items		Mean	Standard deviations	Skewness	Kurtosis
**Anxiety**					
GAD_1	Nervous or anxious	0.82	0.80	0.85	0.39
GAD_2	Uncontrolled worry	0.75	0.85	1.03	0.42
GAD_3	Worry too much	0.91	0.85	0.77	0.15
GAD_4	Trouble relaxing	0.74	0.87	1.03	0.34
GAD_5	Restlessness	0.55	0.78	1.32	1.00
GAD_6	Irritable	0.96	0.87	0.78	0.15
GAD_7	Afraid something awful might happen	0.45	0.74	1.71	2.40
**Depression**					
PHQ_1	Anhedonia	0.95	0.83	0.70	0.09
PHQ_2	Depressed or sad mood	0.70	0.82	1.04	0.46
PHQ_3	Sleep difficulties	0.92	0.93	0.72	−0.43
PHQ_4	Fatigue	1.10	0.86	0.61	−0.05
PHQ_5	Appetite disturbances	0.76	0.84	0.91	0.11
PHQ_6	Feelings of guilt	0.66	0.90	1.23	0.53
PHQ_7	Concentration difficulties	0.65	0.88	1.25	0.69
PHQ_8	Psychomotor agitation or retardation	0.56	0.79	1.37	1.25
PHQ_9	Thoughts of death	0.27	0.58	2.40	5.79
**Fertility related stress**					
	Social concern	31.58	7.47	0.10	−0.03
	Sexual concern	22.32	7.02	−0.46	0.09
	Relationship concern	31.32	6.21	−0.25	−0.23
	Need for parenthood	40.04	8.80	0.10	−0.45
	Rejection of childless lifestyle	33.67	7.48	−0.17	0.38

PHQ-9, Patient Health Questionnaire–9; GAD-7; Generalized Anxiety Disorder-7; and FPI, Fertility Problem Inventory.

In the entire network, “Restlessness (GAD_5)” was the most central symptom across all centrality indices, followed by “Trouble relaxing (GAD_4)” and “Uncontrollable worry (GAD_2)” Following these, “Feelings of guilt (PHQ_6)” was the most central symptom in depression.

### Accuracy and Stability Estimation

**Figure 3:**
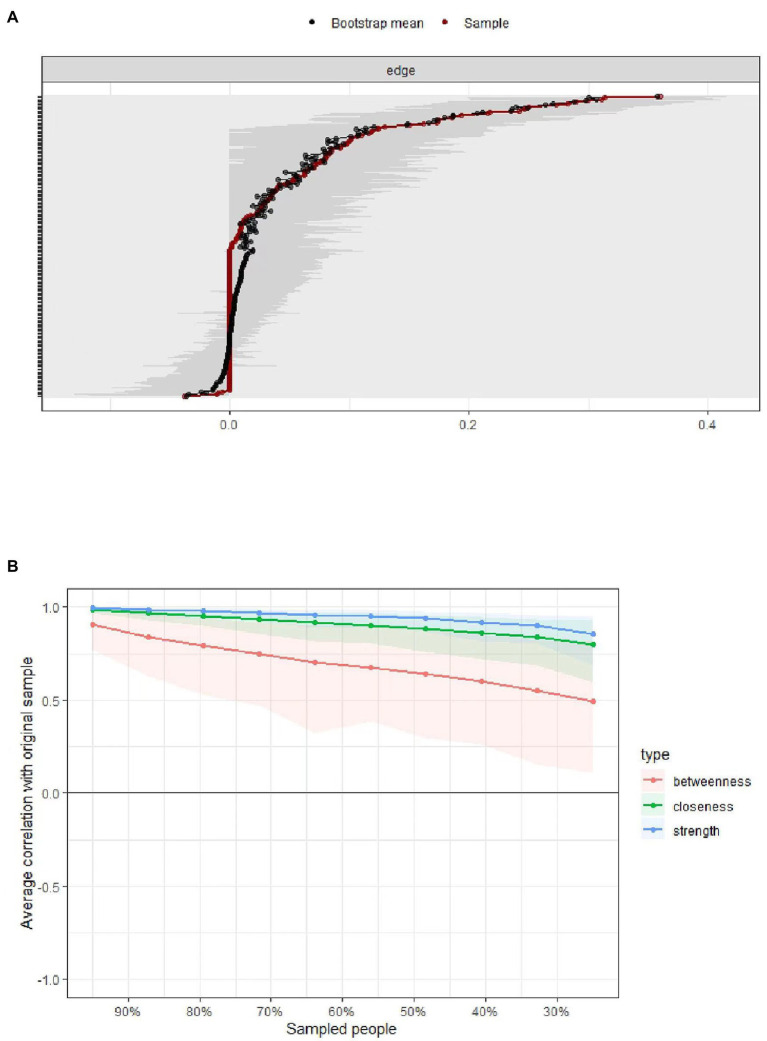
Panel **A**: Bootstrapped confidence intervals (CIs) of the edge weights in the network. The red line indicates the edge weight values, the black line represents bootstrap means, and the gray area the 95% CIs. Panel **B**: Subsetting bootstrap for the network that shows the average correlations between centrality indices of the original network constructed on the full data, with networks estimated on samples with fewer participants.

Accuracy analysis (Figure 3A) showed considerable overlap between the 95% CIs of edge weights, but especially the strongest edges were significantly stronger than many others, which indicated the network was moderately accurately estimated. Stability estimation (Figure 3B) indicated that the order of node strength centrality was more stable than the order of betweenness and closeness. The CS-coefficients for strength, closeness, and betweenness were 0.75, 0.13, and 0.67, respectively. Since node strength was more reliably estimated, the interpretation of findings in this study was based primarily on node strength.

### Sensitivity Analysis

The Network Comparison Test showed that neither network structure (*M* = 0.13, *p* = 0.99) nor global strength (men: 9.38, women: 9.04, *S* = 0.34, *p* = 0.70) differed between the network tested in women and men.

## Discussion

This is the first study to describe anxiety, depression, and fertility-related stress symptom networks in infertility patients using network analysis. Overall, the findings suggest that some symptoms are more correlated than others, and that individual fertility-related stress, depression, and anxiety symptoms are not equally important in the network. Connections within each symptom were higher than connections between symptoms. Neither network structure nor global strength differed between women and men. Even though a growing body of research has focused on depression, anxiety, and fertility-related stress among infertility patients (Volgsten et al., 2010; Omani-Samani et al., 2018; Maroufizadeh et al., 2019), there is limited consensus on the potential ways in which this relationship might be explained. By eliminating or reducing the central symptoms, activity across the network maybe reduced (or prevented).

The edges between “relationship concern stress” and “sexual concern stress” were significantly different from nearly all other edges in the network. Significant edges that appear in the associative network are most likely to constitute true causal links, although our cross-sectional design does not allow for causal inferences. Sex, as an integral relationship and a mutual response between a couple, is supposed to be natural and relaxed. However, once the treatment for infertility begins, sexual life becomes a regular and important task related to therapeutic outcome-pregnancy, and frames of “success” or “failure” in the bedroom tend to kill the more relaxed and sensual frames that may have existed. Infertile couples often talk about the loss of a satisfying sexual life, which may affect their relationship (Hart, 2002). In turn, a poor relationship could affect the quality of sexual life (Kim et al., 2009). Notably, sexual concern stress had a link with the “thoughts of death” symptoms from depression. Sexual problems have been associated with a series of negative health outcomes such as poor marital satisfaction and depression (Schoenfeld et al., 2017; Bai et al., 2019b). In turn, these negative health outcomes were related to suicidal thoughts (Shani et al., 2016). This might explain why sexual concern stress was associated with thoughts of death in this population. Given that it is associated with thoughts of death, sexual concern stress maybe a key form of stress to target in interventions.

The “Afraid something awful might happen” symptom from anxiety and the “Thoughts of death” symptom from depression were the most strongly connected items across anxiety and depression symptoms. This is, to a certain extent, in line with previous qualitative research (Ying et al., 2015) showing that infertility patients equated the period before the results of the pregnancy test were released to waiting for a death sentence. This feeling of anxiety and fear continues until a healthy baby is born successfully. “Afraid something awful might happen” maybe a critical anxiety symptom to target in interventions.

“Restlessness” was the most central symptom of all centrality indices. Other high strength symptoms were the anxiety items “Trouble relaxing” and “Uncontrollable worry.” The PHQ-9 depression item “Feelings of guilt” had the highest strength among PHQ-9 symptoms, which is a hallmark of depression in infertile patients. This result is different from the traditional concept of depression, where depressed or sad mood is a hallmark of depression (Beard et al., 2016). On one hand, infertility patients might examine themselves and their present and past behavior in an attempt to formulate a theory to explain their infertility. They might believe that what they have done or are doing is contributing to their infertility (e.g., previous abortions, sexual behavior, lifestyle, etc.), which thereby leads to guilt (Galhardo et al., 2011). On the other hand, young couples in China are often pressured by their own expectations or those of their parents. There is a Chinese saying that, of all those who lack filial piety, the worst are those who have no children. This traditional value of carrying on the family line is deeply ingrained, and couples often regard having children as a family obligation (Wang et al., 2007; Jin et al., 2013). Thus, childlessness led to sufferers feeling guilty about their families. Notably, “Feelings of guilt” has a strong link with “Thoughts of death,” which is in line with research in major depression disorders (Keilp et al., 2012). The primary link between depression severity and suicidal ideation was *via* sad mood and guilt, not *via* vegetative or somatic symptoms.

Similar to earlier work, our findings are consistent with a model in which symptoms exist in a causally connected network, rather than the equivalent of a common cause disease (Beard et al., 2016). At the most general level, this suggests that past efforts to understand psychosocial distress in infertility patients may have overlooked an important factor in the experience of distress, the causal relationship between symptoms.

The current study suggests several potential developmental interventions to address mental health needs in infertility patients. As mentioned, treatments may be more influential if they target central symptoms. Targeting the anxiety symptoms of restlessness, trouble relaxing, and uncontrollable worry may therefore be most effective in alleviating overall psychological distress. The treatments that first target guilt may be most efficient. Although both antidepressant medication and cognitive behavioral therapy (CBT) are effective in improving depression, an individual patient data meta-analysis suggests that antidepressant medication was more efficacious than CBT in improving guilt (Boschloo et al., 2019). Sexual concern stress, which is associated with suicidal ideation, maybe also a priority target. Providing more sexual health education and social support to infertility patients, and reducing their avoidance responses, may reduce sexually related stress.

Three limitations need to be noted. First, the cross-sectional design did not permit the identification of causal relationships. Experimental and longitudinal designs are needed to examine causal relationships in the future. Moreover, cross-sectional, group-level designs do not necessarily apply to individuals. Individual-level, longitudinal designs are warranted to elucidate individual-level processes. Second, the patients in this study were recruited from one reproductive hospital; especially, most patients had low socioeconomic status, which limits the generalizability of the findings. However, the site of this study is one of only two reproductive hospitals in Ningxia province, serving four regions, including Ningxia, Shanxi, Gansu, and Inner Mongolia. Thus, the sample still contains some broader representation, and is not just one treatment center in a particular area. Third, patients with primary or secondary infertility were all invited to participate in this study, however, we did not collect the information on primary vs. secondary infertility in the sample.

## Conclusion

In summary, this study is the first to describe psychological distress networks in infertility patients using network analysis. Restlessness was the most central symptom in infertility patients. “Feelings of guilt” had the highest strength among PHQ-9 symptoms. “Relationship concern stress” and “sexual concern stress” had the strongest connections in the network. This study reinforces the need to better understand the underlying causes of psychological distress in infertility patients. A more detailed investigation of the relationship between these symptoms could provide information for psychosocial interventions aimed beyond “alleviating psychological distress.” We should consider the individual psychological symptom pattern and its potential causes in infertile patients instead of assuming a consistent psychological distress structure.

## Data Availability Statement

The original contributions presented in the study are included in the article/Supplementary Material, further inquiries can be directed to the corresponding author.

## Ethics Statement

The studies involving human participants were reviewed and approved by ethical review board of the Ningxia Medical University. The patients/participants provided their written informed consent to participate in this study. Written informed consent was obtained from the minor(s)’ legal guardian/next of kin for the publication of any potentially identifiable images or data included in this article.

## Author Contributions

DC analyzed the data and wrote the manuscript. CB designed the study and contributed to data collection. GZ reviewed the manuscript. All authors contributed to the article and approved the submitted version.

## Conflict of Interest

The authors declare that the research was conducted in the absence of any commercial or financial relationships that could be construed as a potential conflict of interest.

## Publisher’s Note

All claims expressed in this article are solely those of the authors and do not necessarily represent those of their affiliated organizations, or those of the publisher, the editors and the reviewers. Any product that may be evaluated in this article, or claim that may be made by its manufacturer, is not guaranteed or endorsed by the publisher.
