# Supplementary material for: Psychological Distress Among Infertility Patients: A Network Analysis
Source: Front Psychol. 2022 Jun 28;13:906226. doi: 10.3389/fpsyg.2022.906226 (PMC9274242; doi:10.3389/fpsyg.2022.906226)
Supplement: Supplementary file 1 [file Data_Sheet_1.pdf]

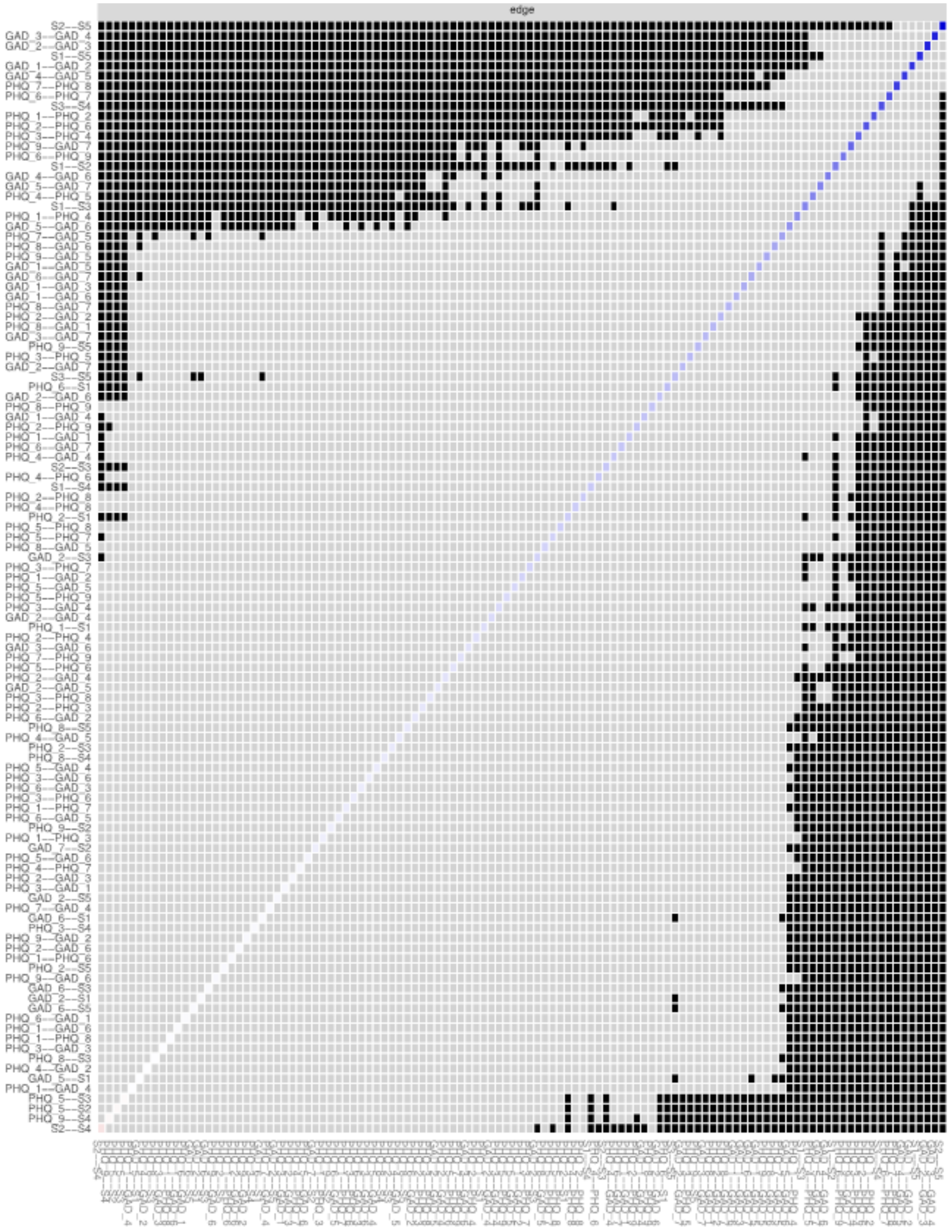

Supplementary Figure 1. Confidence intervals around edges between PHQ-9 (PHQ_1 - PHQ_9), GAD-7(GAD_1 - GAD_7) and FPI (s1 - s5).

Note: PHQ-9, Patient Health Questionnaire–9; GAD-7; Generalized Anxiety Disorder-7; FPI, Fertility Problem Inventory; s1, Social concern; s2, Relationship concern; s3, Need for parenthood; s4, Rejection of childless lifestyle; s5, Sexual concern
